# Fracturing and Damage of 3D-Printed Materials with Two Intermittent Fissures under Compression

**DOI:** 10.3390/ma13071607

**Published:** 2020-04-01

**Authors:** Duan Zhang, Qianqian Dong

**Affiliations:** School of Civil and Transportation Engineering, Hebei University of Technology, Tianjin 300401, China; Dawn950110@outlook.com

**Keywords:** intermittent fissures, fracture, 3D printing materials, crack coalescence, DIC

## Abstract

The crack propagation and failure of 3D-printed samples with prefabricated K–S fissures (a kinked fissure and a straight fissure) were observed under uniaxial compression, and the strain and displacement of the sample surface were quantified by the digital image correlation (DIC) method. The experimental results show that the branch inclination angle of the kinked fissure is an important factor affecting the crack initial position, and the evolution of the strain field during the failure process of the sample can better reflect the cracking law of the internal fissures. Furthermore, two coalescence modes are classified: Mode I is a tension–shear composite failure formed by the penetration of the tension–shear composite crack; Mode II is a tensile failure that penetrates the whole samples during the failure process without rock bridge damage. In addition, the numerical simulation results were well consistent with the cracking and failure modes.

## 1. Introduction

For a preexisting fissure, new cracks form and subsequently coalesce with other preexisting fissures due to a disturbance, which causes the failure of the rock bridge and eventually causes overall damage [1,2,3,4,5]. Therefore, the failure mode of the rock bridge between fissures plays an essential part in predicting the failure process of preexisting fissures when they are subjected to external load. Many scholars have conducted experimental studies [6,7,8,9] and numerical analysis [10,11,12] on the crack propagation and failure behavior of the rock bridge for samples containing prefabricated straight fissures. On the other hand, theoretical [13,14,15,16] and experimental [17,18,19] studies on non-straight fissures mainly focused on the stress intensity and the crack propagation path of a single fissure. However, the failure mode of rock bridges under the interaction of a straight fissure and a non-straight fissure has not been fully elucidated.

Some previous experiments have investigated the failure characteristics of brittle materials with multiple straight fissures. Sagong and Bobet [6] carried out compression experiments on samples containing two to 16 fissures, and the results indicated that the crack propagation in the multiple-fissure samples was similar to those that contained two fissures. The two-fissure cases have been experimentally studied by Wong and Einstein [7], whose results indicated that the fissure inclination angle, bridging angle, and ligament length have a strong influence on the crack coalescence. Huang et al. [8] conducted triaxial compression tests on samples with two closed non-overlapping flaws and found that the arrangement of the flaw pair had a more significant influence on the rock deformation, rock strength, and crack combination mode than the confining pressure. Yang et al. [9] performed a triaxial compression test on a sandstone sample containing two preexisting 3D defects, which showed shear crack coalescence in the ligament region and identified indirect coalescence outside the ligament region.

**Figure 1:**
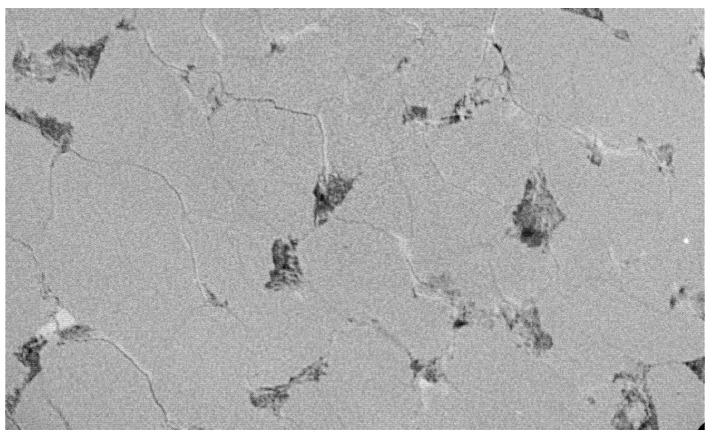
YXLON FF35 CT scan of a granodiorite sample.

Non-straight fissures extensively exist in natural environments. Figure 1 shows a natural granodiorite sample under CT scanning, where many non-straight fissures can be observed. In the past, some scholars have also performed theoretical and experimental research on non-straight fissures. Isida and Noguchi [13] gave a reliable formula for the stress intensity factors of various branch cracks and calculated the stress intensity factors of all the branches and the main crack tip. Li et al. [17] performed a compression test on a cracked marble sample containing prefabricated circular holes and concluded that the direction and geometry of the cracks determine where the secondary cracks will occur. Meggiolaro et al. [15] proposed crack retardation equations to numerically estimate the crack path and associated stress intensity factors of kinked and bifurcated cracks. Carpinteri et al. [16] quantified the fatigue crack growth rate of periodic kinked cracks and made a comparison with that of ordinary cracks, which suggested that the kinked rate decreases as the kinked angle increases, and the fatigue cracks propagated, whose velocity is related to the kinked angle and the crack length. Recently, Ma et al. [18] performed uniaxial compression experiments on straight (S)-shaped fissures. It was found that the effective curvature has a strong influence on the crack initial position. A single kinked fissure was also investigated by Ma et al. [19], whose results have shown that the tensile crack propagation of wing cracks is the major type of failure of kinked fissures under the uniaxial compression experiments. Double S-shaped fissures in natural marble specimens were investigated by Dong et al. [20]. It was found that the decrease of strength is accompanied by the internal crack at the stage of crack growth and propagation, following the occurrence of the external crack, at relatively large flaw and ligament angles. However, for intermittent non-straight fissures, especially the effect of the interaction between intermittent K–S fissures (a kinked fissure and a straight fissure) on their behavior patterns in cracking, the propagation and coalescence behaviors under experimental conditions have not yet been sufficiently discussed.

The experimental materials can be categorized into rock-like materials [21,22,23] and actual rock materials [24,25,26]. The traditional methods for producing prefabricated fissures include inserting a sample with a mica sheet or a steel sheet [27] and cutting by a water-jet machine [21] or high-precision electric cutting machine [28]. The 3D printing technology has advantages over the above traditional manufacturing methods for its efficiency, accuracy, and repeatability. The adopted 3D printing material is resin consisting of a rapidly curing epoxy prototype compound, which is brittle and linearly elastic under freezing conditions and is free of residual stress. In addition, the excellent transparency of such material makes it possible to observe the crack propagation inside the material. Thus, it has been extensively employed by scholars. For example, Ju et al. [29] used the 3D printing technique to generate a physical model representing natural coal rock that contains complex fractures for uniaxial compression tests. It was found that the location of the stress concentration and the stress gradient around the discontinuous fractures are in good agreement with the numerical predictions of the real coal sample. Zhou et al. [30] performed uniaxial compression tests on samples containing single and two defects by employing 3D printing technology, and the tests showed that the defect geometry significantly affected the mechanical properties and fracture behavior of the defect samples.

In addition, numerical simulation methods are also widely used in fracture mechanics, and they have effectively overcome the shortcomings in crack propagation research. Commonly used numerical simulation methods include the finite element method [31,32,33], boundary element method [34], discrete element method [35], numerical manifold method [36], displacement discontinuity method [37], and finite difference method [38]. These methods have been used to simulate the initiation, propagation, and coalescence process of cracks. Among these methods, the finite element method is a mature calculation method, and some software applications based on this method. For example, the software RFPA^2D^ produced by Tang et al. [39] has been recognized and applied in the research of crack propagation, the numerical simulations of non-straight fissures are in good agreement with the experimental results [17]. 

Therefore, a photosensitive resin material was used for 3D printing, and samples containing K–S fissures were prepared. Six inclination angles of the branch in the kinked fissure were selected, and uniaxial compression loading of these samples was performed using a rock mechanics servo-controlled testing system. As a result of the transparency of the samples, the crack initiation and propagation process of prefabricated fissures can be clearly observed. In addition, digital image correlation (DIC) technology was used to monitor the evolution of the strain during the whole test. The strength, crack initiation, and propagation process of the sample and the coalescence of the rock bridge between prefabricated fissures were studied. Finally, the samples were numerically simulated using RFPA^2D^ software.

## 2. Sample Preparation and Testing

### 2.1. 3D-Printed Sample Preparation

**Figure 2:**
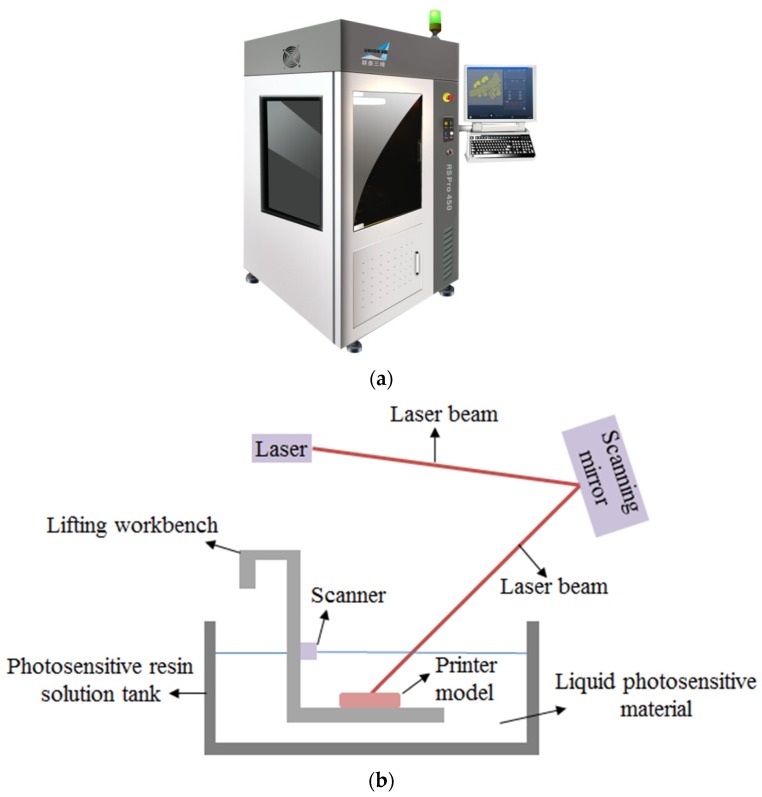
3D printer and the principle of stereo lithography appearance (SLA). (a) Diagram of 3D printer (the type is RS Pro 450); (**b**) Schematic diagram of stereo lithography apparatus.

The experimental samples were produced by the RS Pro 450 3D printer whose forming range is 450 × 450 × 350 mm^3^ (L × W × H), as shown in Figure 2a. A laser curing rapid prototyping 3D printing technology, the stereo lithography appearance (SLA), is used, and the principle is as shown in Figure 2b. First, the laser scans selectively depending on the shape of the cross-section of the samples. The photosensitive resin material is printed in the relevant area of the cross-section and solidified under the irradiation of the laser. Then, the lifting workbench of the photosensitive resin solution tank decreases along the Z-axis to a certain height, and the next layer is printed and solidified by the laser. Then, the printer prints and solidifies layer by layer until the samples are completed. Finally, the samples are obtained by removing the residual photosensitive resin on the surface of the samples. The scanning speed of the device is 6–10 m/s, the dot precision is 0.02–0.1 mm, and the printing thickness is between 0.05 and 0.25 mm. Table 1 lists the basic mechanical properties of the 3D-printing material, and Table 2 lists the basic mechanical properties of 3D-printed materials after cryogenic freezing.

**Figure 3:**
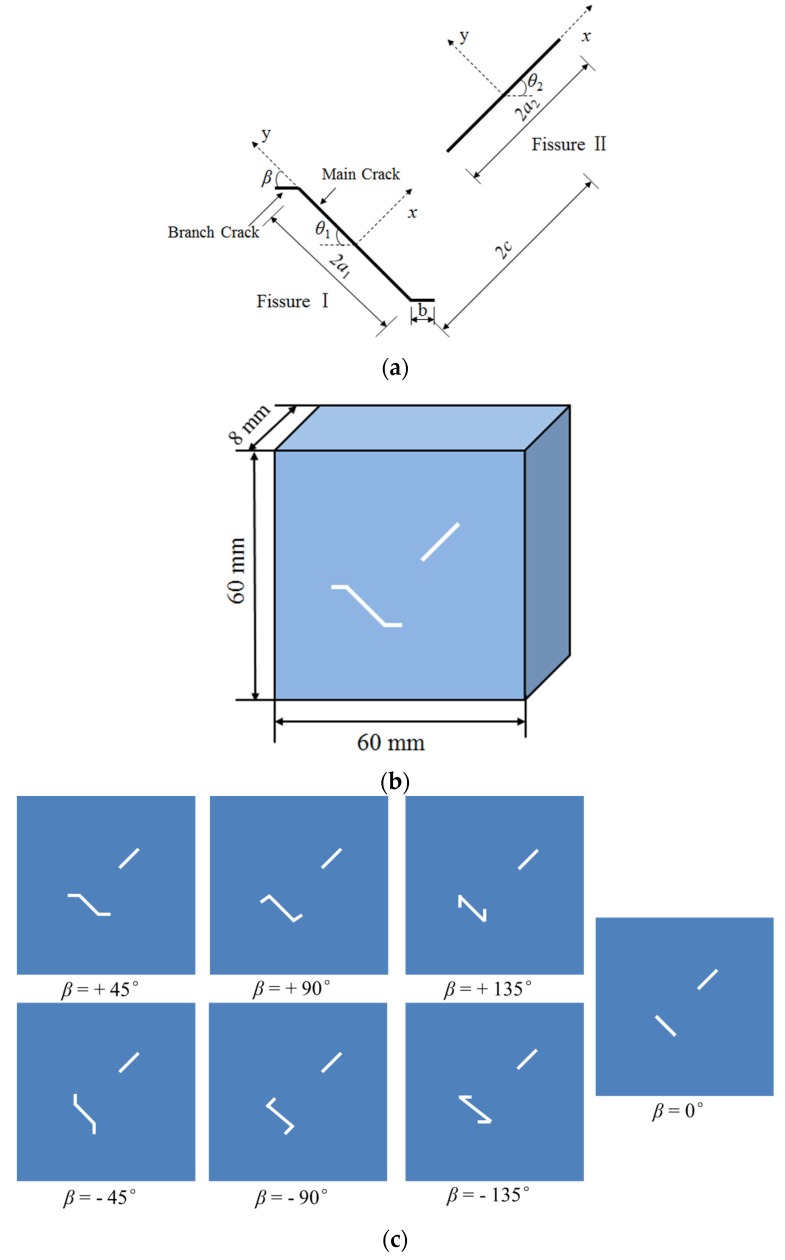
Schematics of fissures in the samples. (**a**) Sketch of geometries of fissures; (**b**) Diagram of one model; (**c**) Different geometry configurations of fissures in the samples.

Fissure I is a kinked fissure, and fissure II is a straight fissure, as illustrated in Figure 3a. The geometric arrangements of fissure I and fissure II are determined by seven geometrical parameters: the main fracture length of fissure I, 2*a*_1_, the branch fracture length of fissure II, *b*, the main fracture inclination of fissure I, *θ*_1_ (the angle between fissure I and the horizontal axis), the branch fracture inclination of fissure I, *β* (the angle between fissure II and the y-axis), the length of fissure II, 2*a*_2_, the inclination of fissure II, *θ*_2_ (the angle between fissure II and the horizontal axis), the distance between kinked fissure I and straight fissure II, 2c; and the two fissures are coplanar and nonparallel. As shown in Figure 3b, each sample had a height of 60.0 mm, a width of 60.0 mm, and a thickness of 8.0 mm. 

In these samples, different branch fracture inclination angles were designed by changing *β* while keeping the other six parameters unchanged (2*a_1_* = 2*a*_2_ = 6 mm, *θ*_1_ = −45°, *θ*_2_ = + 45°, *b* = 2 mm, and 2*c* = 14.1 mm), as shown in Figure 3c, and the branch fracture inclination angle, *β*, is designed as +45°, +90°, +135°, −45°, −90°, and −135°. In addition, there is a control group containing two nonparallel straight fissures. Each sample type includes four identical samples, and a detailed description of the samples containing different geometry fissures is presented in Table 3. To avoid the experimental results being affected by the boundary effect of the sample, the two prefabricated fissures were placed at positions distant from the side boundaries of the samples.

It is important to note that the term “fissure” is used to describe a prefabricated artificial defect, and the term “crack” is used to describe new fractures and damages formed under loading.

### 2.2. DIC Equipment Preparation

The DIC technique is a widely used full-field measurement technique. Conceptually, the DIC technique is only a particle tracking method that can be used to determine the position of particles (spots) in digital images. More specifically, DIC is a non-contact optical method for analyzing digital images to extract the field displacement surface of specimens [38]. DIC can determine displacement and strain fields in different scales without touching the observed surface and has the real-time performance that can measure over the full field of vision and over a wide field [39]. The DIC equipment includes cameras, tripods, and image processing systems. In previous studies, many scholars have applied the DIC to the measurement of stress intensity factors [40,41,42], determined the occurrence of fractures and the evolution of crack lengths [43,44], and conducted multiscale research [45,46].

### 2.3. Testing Procedure

**Figure 4:**
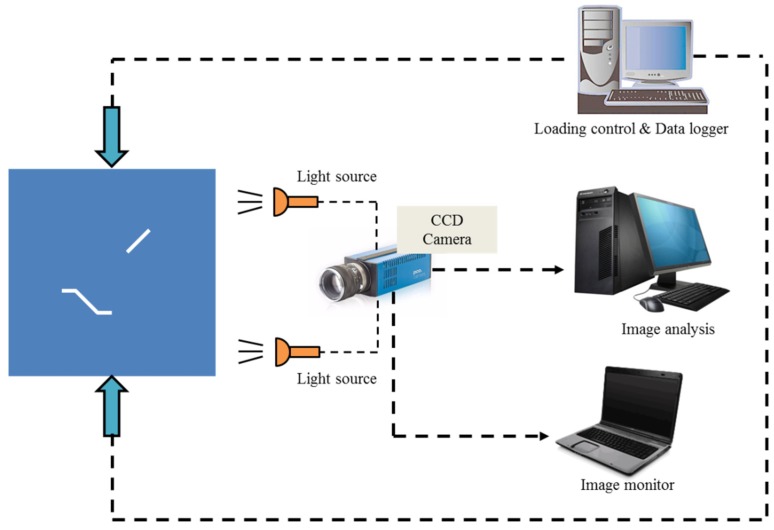
Loading system and digital image correlation (DIC) equipment under uniaxial compression.

Uniaxial compression tests of the photosensitive resin samples containing intermittent K-S fissures were conducted using a rock mechanics servo-controlled testing system, as shown in Figure 4. Each sample is placed between the two loading platforms. To reduce the end effects of specimens produced during test loading and reduce their impact on the experimental results, petrolatum is applied between the rigid head of the test machine and the contact surface of samples. Thus, the effect of the confining pressure caused by friction is avoided as much as possible. The axial stress to the two surfaces of the sample is increased until the sample fails. All samples were loaded at a constant displacement rate of 0.5 mm/min under displacement control conditions until the samples fail. High-speed cameras are used to monitor the entire process of the samples from the beginning of loading to failure. The type of high-speed camera is a Photron SA1.1, and image acquisition rates range from 1000 to 1,000,000 images per second, with a maximum resolution of 4 megapixels. Furthermore, based on the DIC technique, the full-field displacement and strain before and after the failure of the samples were studied, as shown in Figure 4.

## 3. Experimental Results and Discussion

### 3.1. Certification of the Failure Mode

**Figure 5:**
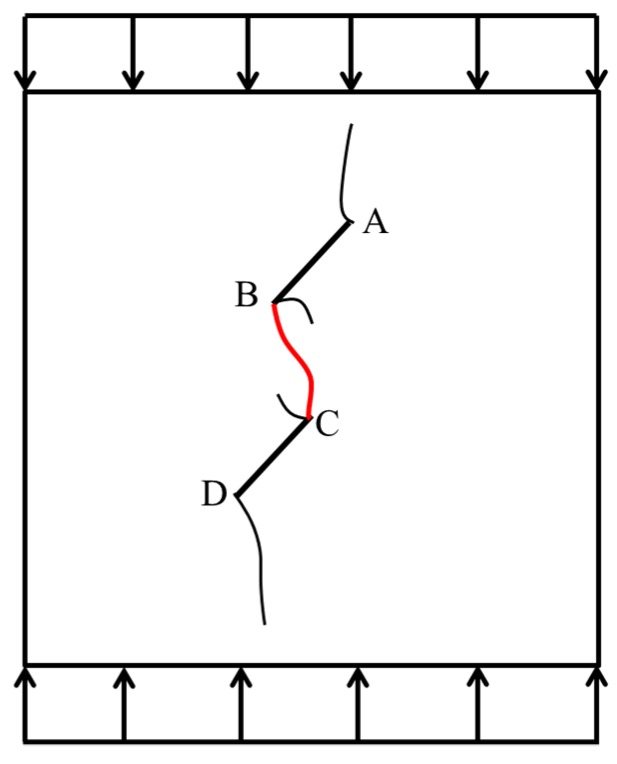
Tension–shear composite failure of rock bridge.

The tensile–shear composite failure of the rock bridge is caused by the coalescence of a tensile–shear composite crack in the rock bridge. As shown in Figure 5, a tensile–shear composite crack BC penetrates the rock bridge that between the prefabricated fissure AB and fissure CD. The coalescence strength of the rock bridge is estimated according to the following assumptions [47,48]:

(1) The development of tensile–shear composite cracks is in the direction of σ1, and the normal stress on the surface reaches the tensile strength σt of the material.

(2) The stress state on the tensile–shear composite crack surface meets the Mohr–Coulomb criterion.

Then, the coalescence strength of the rock bridge is

(1) $$\sigma {\;}_{1}=\frac{h\sigma_{t}(\sin\alpha +f_{r}\cos\alpha )-4lc_{r}}{A}\;$$

(2) $$\begin{array}{l} A=-(4a\sin\varphi +4l\sin\alpha )(-f_{r}\sin\alpha +\cos\alpha )+2aC_{t}\sin(2\varphi )\\ \left[-f_{r}\sin(\alpha -\varphi )+\cos(\alpha -\varphi )]-4aC_{n}\sin^{2}\varphi [f_{r}\cos(\alpha -\varphi )+\sin(\alpha -\varphi )\right] \end{array}$$

(3) $$l=\frac{h_{0}}{\cos\varphi }.$$

Among them, *h* *(mm)* is crack horizontal spacing, *σ*_t_ *(N/mm2)* is the uniaxial tensile strength of the sample material, *α* (º) is the inclination angle of the rock bridge, *φ* (º) is the angle between *σ*_1_ *(N/mm2)* and the crack, *f*_r_ is the coefficient of friction, *c*_r_ *(N)* is the cohesion, *l* *(mm)* is the propagation of the branch crack, *h*_0_ *(mm)* is the vertical distance between two cracks, *C*_n_ is the pressure transfer coefficient, and *C*_t_ is the shear transfer coefficient.

**Figure 6:**
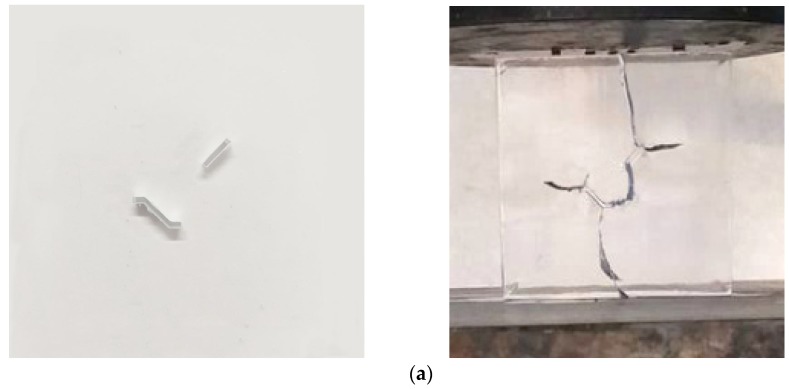

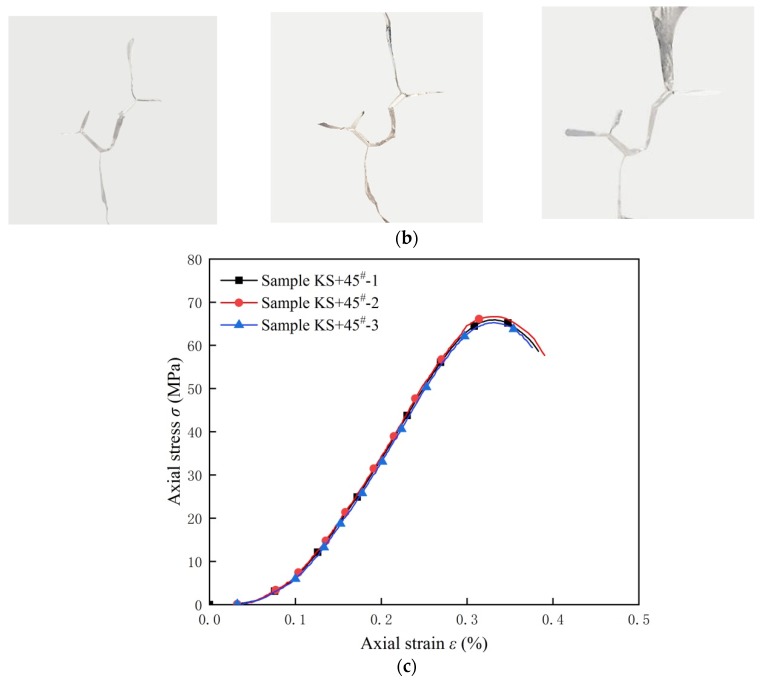
Before and after the experiment of sample KS + 45^#^. (**a**) Photos of the sample before and after compression; (**b**) Failure mode of three samples; (**c**) The stress–strain curves of three samples KS + 45^#^-1, KS + 45^#^-2, and KS + 45^#^-3.

Figure 6a shows the sample KS+45^#^ before and after the experiment, and the consistency of failure mode for samples KS + 45^#^-1, KS + 45^#^-2, and KS + 45^#^-3 after the experiments is shown in Figure 6b. In addition, the stress–strain curves of samples KS + 45^#^-1, KS + 45^#^-2, and KS + 45^#^-3 is shown in Figure 6c, and their strengths were similar. The stress–strain curve consists of four typical main phases. The strain of the sample increases with the increase of the stress at the beginning of the experiments. The stress–strain curve shows a nonlinear change and a slight upward concavity at low stress levels. This phase can be referred to as the initial compaction phase. After the initial compaction phase, the stress–strain curve increases linearly, in a manner similar to Hooke’s law, and this phase can be called the elastic deformation phase. The elastic modulus of a sample is the slope of the stress–strain curve in this phase, and it remains constant. With increasing applied load, the samples show nonlinear hardening behavior, which can be called the nonlinear deformation phase. A continuous stress concentration near the crack tip that occurs in this phase causes a crack to initiate and propagate, and new cracks are formed thereafter. With further loading, a sudden drop in the axial stress of the sample occurs beyond the peak strength, and the sample fails macroscopically. This phase can be referred to as the macroscopic failure phase.

Therefore, the 3D-printed resin material is stable and can be used to study the macro failure mode of the brittle material.

### 3.2. Analysis of the Axial Stress–Strain Curves of Specimens

**Figure 7:**
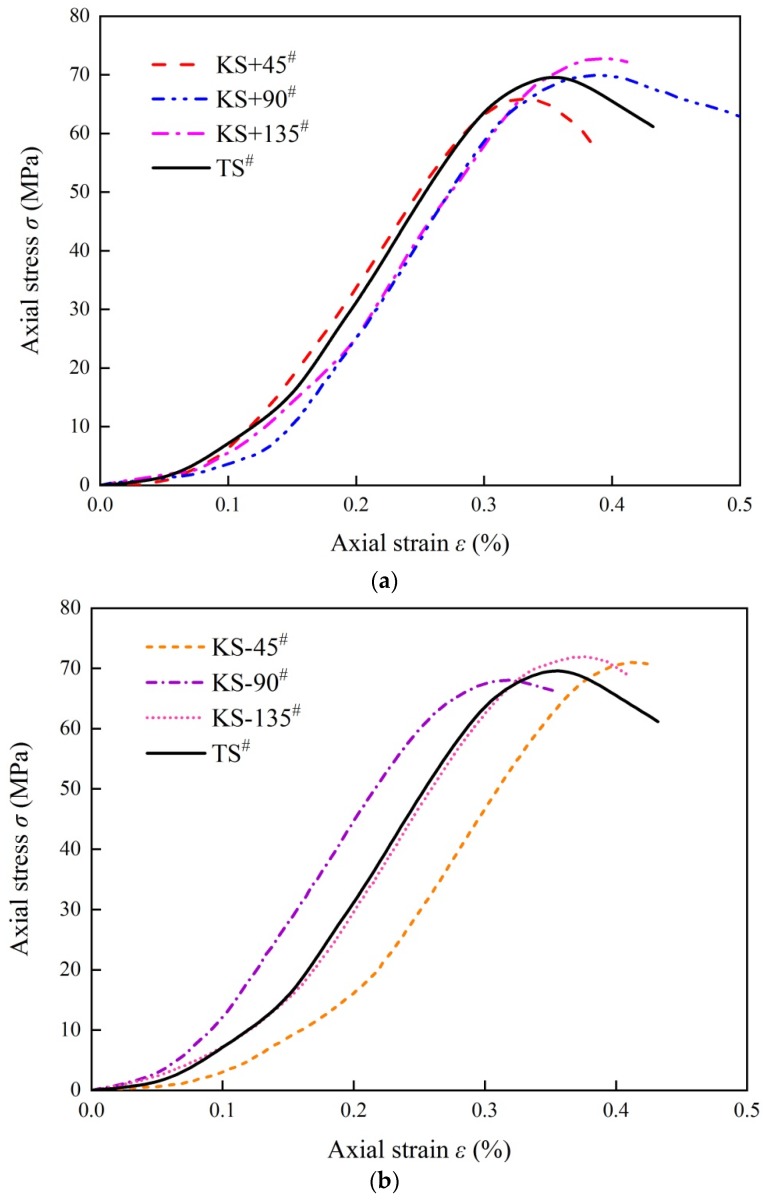
Axial stress–strain curve of different samples under uniaxial compression: (**a**) Stress–strain curve of changing the inclination angle *β,* rotating counterclockwise; (**b**) Stress–strain curve of changing the inclination angle *β*, rotating clockwise.

Figure 7 shows the axial stress–strain curves of the two sets of samples under uniaxial compression, and each curve is fitted by three curves measured by three identical samples. Sample TS^#^ contains two intermittent straight fissures. Samples KS + 45^#^, KS + 90^#^, KS + 135^#^, KS - 45^#^, KS - 90^#^, and KS - 135^#^ all contain a kinked fissure and a straight fissure, and the branch fracture inclination angles are +45°, +90°, +135°, −45°, −90°, and −135°. According to Figure 7a,b these samples represent shapes similar to that of the sample containing only straight fissures. 

Figure 7a exhibits the average peak strength of the axial stress–strain curves of the four samples. Sample TS^#^ exhibits an average peak strength of 69.58 Mpa, and for the samples with kinked fissures whose angles are positive, the average peak strength values are not very different from that of sample TS^#^, 65.90 Mpa (KS + 45^#^), 69.92 Mpa (KS + 90^#^), and 72.77 Mpa (KS + 135^#^). The peak strength of sample KS + 45^#^ decreased by 5.58%, the peak strength of sample KS + 90^#^ exhibited almost no significant change compared with that of sample TS^#^: it only increased by 0.49%, while the peak strength of sample KS + 135^#^ increased by 4.38%. The maximum strength (KS + 135^#^) differs from the minimum strength (KS + 45^#^) by approximately 9.96%. Figure 7b shows that the average peak strength values of the axial stress–strain curves of the three samples containing kinked fissures are 70.98 Mpa (KS - 45^#^), 68.09 Mpa (KS - 90^#^), and 71.93 Mpa (KS - 135^#^). The peak strength of sample KS - 45^#^ increased by 1.97% compared with that of sample TS^#^, while that of sample KS-90^#^ decreased by 2.19%, and that of sample KS-135^#^ increased by 3.27%. The maximum strength (KS - 135^#^) differs from the minimum strength (KS - 45^#^) by approximately 5.64%.

In conclusion, when the inclination angle of the branch of the kinked fissure rotates clockwise, the peak strength increases, and the difference between the maximum strength and the minimum strength is approximately 9.96%. When the inclination angle of the branch of the kinked fissure rotates anticlockwise, the peak strength will not change obviously, and the difference between the maximum strength and the minimum strength is approximately 5.46%.

### 3.3. Cracking Process Based on the DIC Analysis

**Figure 8:**
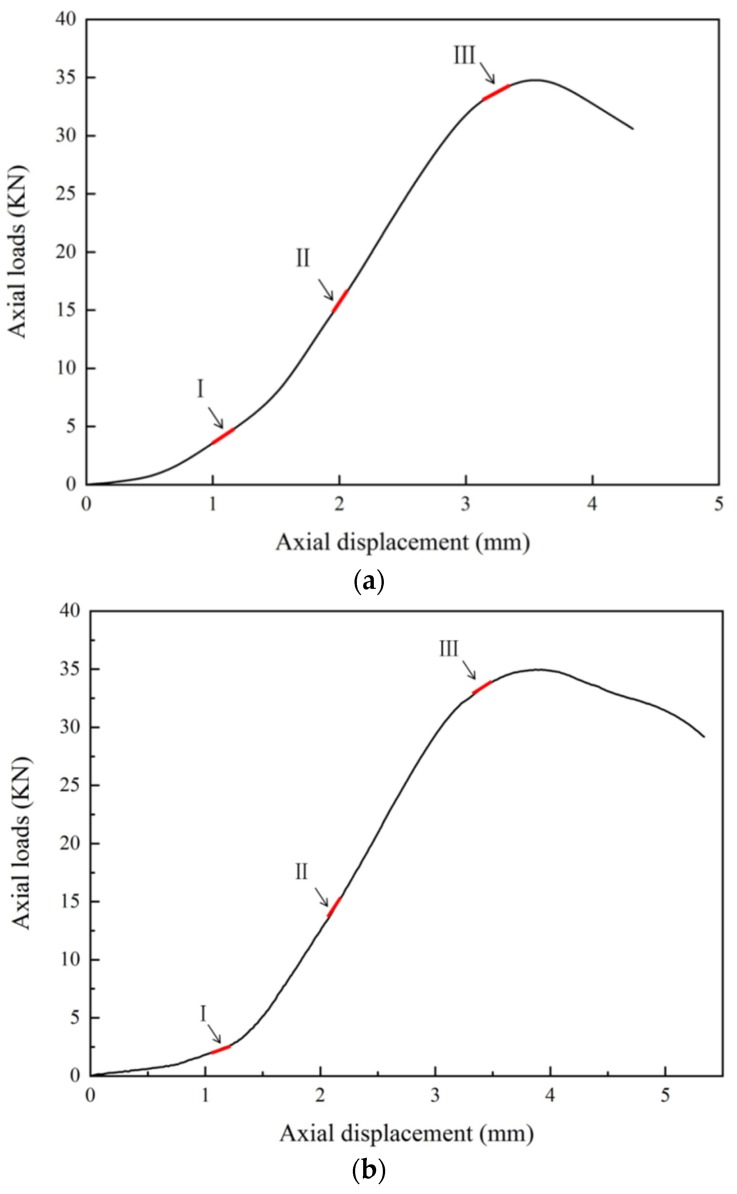

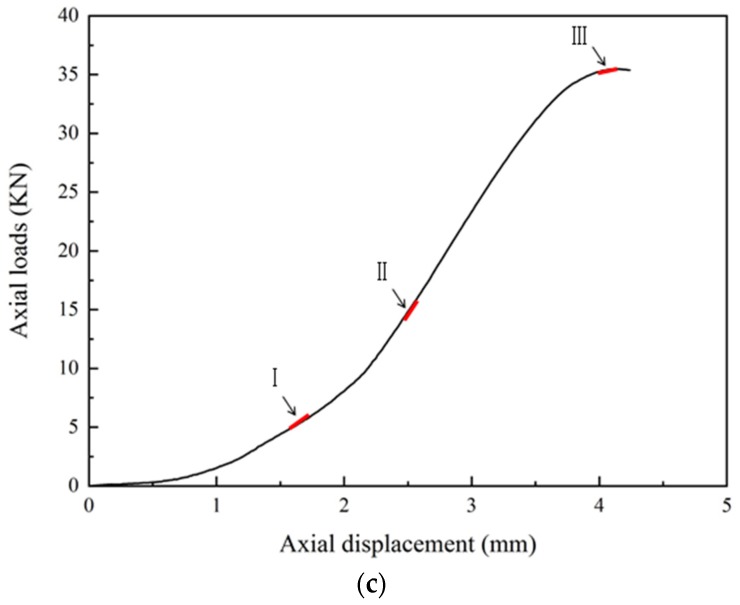
Load–displacement curves for experiments. (**a**) TS^#^; (**b**) KS + 90^#^; (**c**) KS - 45^#^.

**Figure 9:**
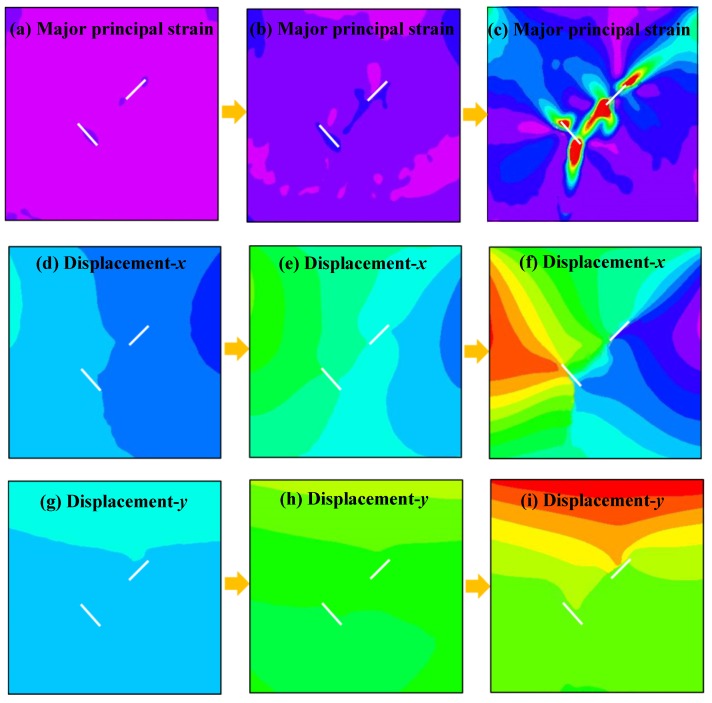
Major strain field and displacement field of sample TS^#^ measured by DIC.

**Figure 10:**
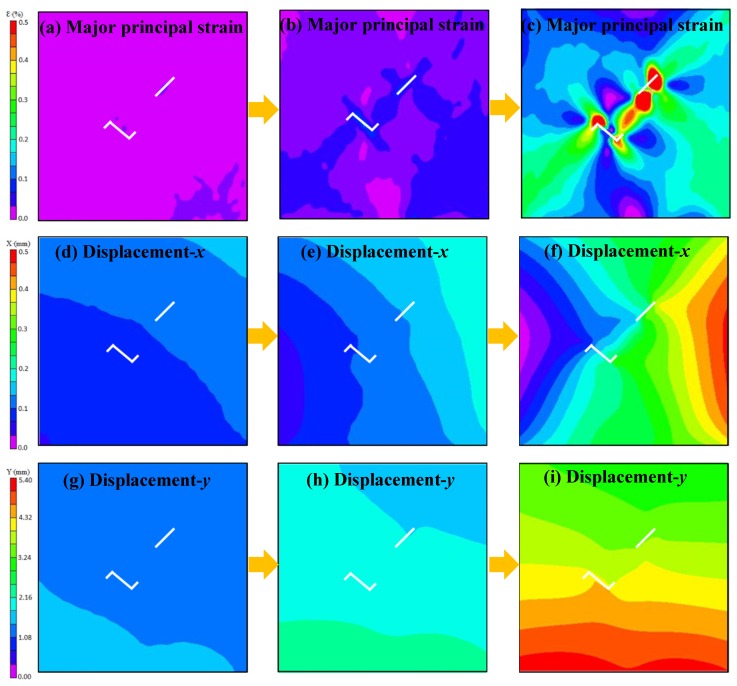
Major strain field and displacement field of sample KS + 90^#^ measured by DIC.

**Figure 11:**
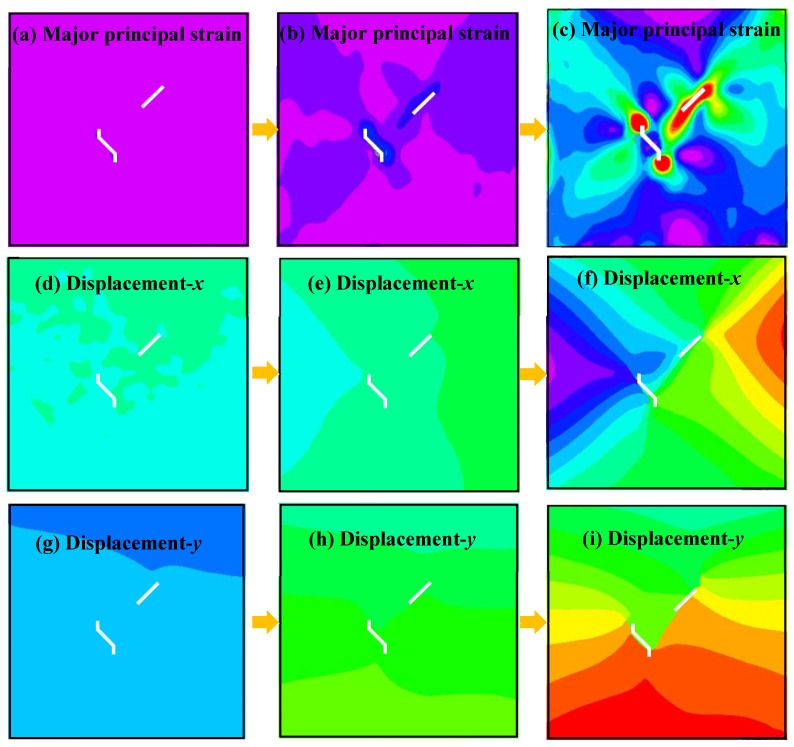
Major strain field and displacement field of sample KS - 45^#^ measured by DIC.

Figure 8 shows the relationship between the axial load and axial displacement of the loading equipment. In the whole loading process of a single sample, approximately 400 photographs of the sample surface were taken by the DIC camera. According to the types of crack propagation and failure mentioned above, three samples, TS^#^, KS + 90^#^, and KS - 45^#^, were selected as the analysis cases. Three photos of three stages in each sample were selected for DIC analysis. These three phases are marked as I, II, and III. Phase I corresponds to the beginning of the loading stage, phase II corresponds to the stage of sample cracking, and phase III corresponds to the stage before the peak stress. The results of the DIC analysis are shown in Figure 9, Figure 10 and Figure 11. The a, b, and c subfigures of each group of pictures correspond to the evolution of the principal strain; the d, e, and f subfigures correspond to the change of the displacement field in the x-direction; and the g, h, I subfigures corresponds to the change of the displacement field in the y-direction. 

In the cracking process of sample TS^#^, during Phase I, there is some strain shrinkage at the edge of the sample and near the prefabricated fissure, while the principal strain of the whole sample is almost uniform in all regions (Figure 9a). The maximum value of displacement in the x-direction is on the right side (Figure 9d), and the maximum value of displacement in the y-direction is on the upper side (Figure 9g). As the loading continues, during Phase II, stress concentrations first appear around the prefabricated fissure in the strain field and then extend (Figure 9b). There are some gradient changes in the x-direction displacement (Figure 9e) and the y-direction displacement (Figure 9h), but these changes are small, indicating that the displacement in these two directions does not show large deformation. Finally, during Phase III, serious strain concentration occurs in the crack tips of the two straight fissures and the rock bridge between the two straight fissures (Figure 9c). The displacement values increase continuously, and the displacement field shows obvious gradient changes and discontinuities (Figure 9f, and Figure 9i). According to the DIC analysis, it can be concluded that failure first occurs at the crack tips of the two prefabricated straight fissures, which corresponds to the cracking mode of the straight fissures noted in Table 4. With the increase of the axial load, the two prefabricated straight fissures tend to coalesce at the rock bridge.

In the cracking process of sample KS + 90^#^, the principal strains of the whole sample are almost uniform at the beginning of loading. Serious stress concentrations occur at both crack tips of the straight fissure and the inflection points of the kinked fissure, and stress concentration also occurs in the rock bridge when the fissures are cracking. Apparent displacement gradient changes and discontinuities appear in the displacement field, as shown in Figure 10. In contrast, serious strain concentrations occur at the crack tips of the prefabricated straight fissure and kinked fissure when the sample KS - 45^#^ is cracking, as shown in Figure 11.

Therefore, DIC technology can effectively identify the position of the initial crack of prefabricated fissures, and the evolution of the strain field and displacement field during the failure of a sample can better reflect the cracking and propagation of the internal crack.

### 3.4. The Crack Propagation and Failure Behavior

To study the crack propagation and failure behavior of samples containing K–S fissures under uniaxial compression, the first crack is defined as crack 1, the second crack is defined as crack 2, the third crack is defined as crack 3, and the mode of crack 1 is defined as the cracking mode. When the compressive load reaches a certain level, cracks occur in the photosensitive resin samples and then propagate steadily. Within 3 s after the peak stress, all samples fail. Clear cracking sounds can be heard during the tests, and in some samples, the cracking is accompanied by surface resin fragmentation and shedding. All the samples show a certain degree of plastic deformation after failure, and since the samples are transparent, the plastic deformation is not obvious in the photograph. 

It can be observed in Table 4 that the cracking positions of the straight fissures in all samples are almost all at the same position, and that two tensile cracks are formed from the two tips of the straight fissures that propagate to the top and bottom edges of the samples. Furthermore, the tensile crack formed at the upper right of the straight fissure and the lower left of the kinked fissure extends in the direction of the maximum principal stress and then coalesce throughout the whole sample. The cracking position (the position where crack 1 is generated) of the kinked fissures has a large difference from the cracking position of the straight fissures. In samples KS + 45^#^, KS + 90^#^, and KS + 135^#^, the cracks are formed at the inflection point of the whole linked fissure, but in samples KS - 45^#^, KS - 90^#^, and KS - 135^#^, cracks are formed at the two end points of the kinked fissure. As is the case of the tensile crack of the straight fissure, the two tensile cracks of the kinked fissure extend and expand continuously the top and bottom of the specimen, that is, in the direction of the maximum principal stress. As the load increases, secondary cracks are generated and propagated, forming many types of cracks. In addition, the crack types of the samples are shown in Table 4, in which T is defined as a tensile crack, L is a secondary transverse crack, S is a shear crack, and Ss represents surface spalling.

At the same time, the rock bridges of some samples are coalesced and destroyed. Two patterns of failure mode were observed in these experiments, a tension–shear composite failure pattern occurred in the rock bridge is defined as in mode I, and the pattern of tensile failure of the whole sample with no coalescence of the rock bridge is defined as mode II. Samples exhibiting mode I failure include TS^#^, KS + 45^#^, KS + 90^#^, KS + 135^#^, and KS - 135^#^, and the rock bridges of these samples were broken through by tensile–shear composite cracks. Samples exhibiting mode II include KS - 45^#^ and KS - 90^#^, and no coalescence failure occurred in the rock bridge between two fissures in each sample, but only tensile failure occurred throughout the samples.

For the kinked fissures in the samples KS + 45^#^, KS + 90^#^, and KS + 135^#^, the cracked branch fissures are on the right side of the vertical direction and close to the straight fissures tip. When the straight fissure tip initiates and propagates along the direction of the maximum principal stress, the branch fissure tends to be connected to it. In samples KS - 45^#^ and KS - 90^#^, the cracked branch fissures are on the left side of the vertical direction and far from the straight crack tip, so they initiate in the direction of the maximum principal stress without penetration. In particular, for the sample KS-135#, although the branch fissure initiated is vertically to the left and away from the tip of the straight fissure, because it is a horizontal branch, an acute angle is formed at the branch fissure inflection point to cause stress concentration. Therefore, the crack propagation at the straight fissure tip has a tendency to coalesce the inflection point of the kinked fissure.

**Figure 12:**
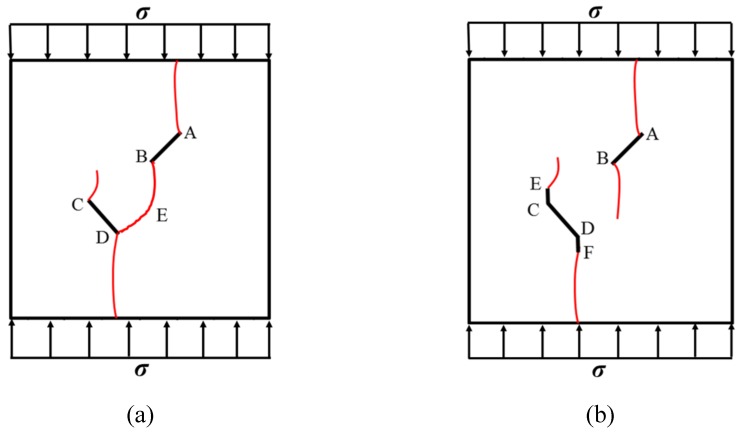
Two failure modes of experiments. (**a**) Mode I with rock bridge tension–shear composite coalescence; (**b**) Mode II without rock bridge damage.

In conclusion, when β = +45°, +90°, and +135°, the kinked fissures all originate from the non-crack tip (the inflection point), and when β = −45°, −90°, and −135°, the kinked fissures all originate from the crack tip. Two failure modes were identified, as shown in Figure 12. Mode I was a tensile–shear composite failure that occurred at the rock bridge between two prefabricated fissures. Mode II was a tensile failure that penetrated the sample up and down without rock bridge failure.

## 4. Numerical Simulations 

Numerical calculations of the crack propagation of samples with straight fissures and K–S fissures were performed using RFPA^2D^ software. The calculation method is based on the finite element theory and statistical damage theory and considers the heterogeneity of the material properties and the randomness of defect distribution. 

The width and the height of the numerical model are both 60 mm, and a square element grid was used to divide the sample into 200 × 200 = 40,000 square elements. The parameter settings are shown in Table 5. The static analysis takes advantage of a plane strain model, and the initial material is solid. The Mohr–Coulomb criterion is used as the strength criterion for this uniaxial compression test. The single-step increase is 0.0001 mm, and the total number of loading steps is 80.

**Figure 13:**
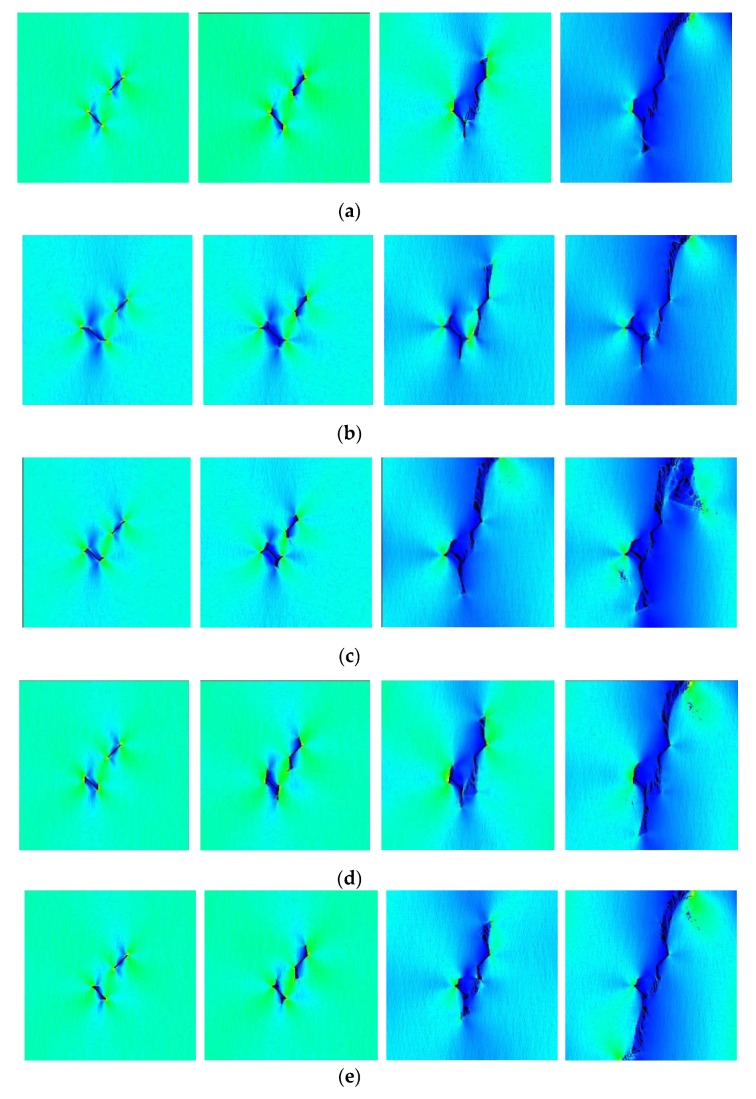
Rock bridge coalescence mode I. (**a**) Crack propagation process of sample TS^#^; (**b**) Crack propagation process of sample KS + 45^#^; (**c**) Crack propagation process of sample KS + 90^#^; (**d**) Crack propagation process of sample KS + 135^#^; (**e**) Crack propagation process of sample KS - 135^#^.

Figure 13a shows the crack propagation patterns of sample TS^#^. At the beginning of the loading, a significant stress concentration occurred at the prefabricated fissures tips. When the loading was continued for 20 steps, the crack tips of the straight fissures initiated and formed tensile cracks. When the load continued to step 50, the tensile cracks extended along the axial direction. During the development of the tensile cracks, the tips produced a stress concentration zone, and the stress concentration at the tips further guided the propagation of the tensile cracks for step 60. In addition, small cracks appeared at the position of the rock bridge. At step 80, which is the end of the loading, the rock bridge coalesced, two tensile cracks penetrated through the whole sample, and the sample failed. Figure 13a–d shows the cracking and propagation of samples KS + 45^#^, KS + 90^#^, KS + 135^#^ and KS-135^#^ are similar to that of sample TS^#^, and the rock bridges of these samples were penetrated. 

**Figure 14:**
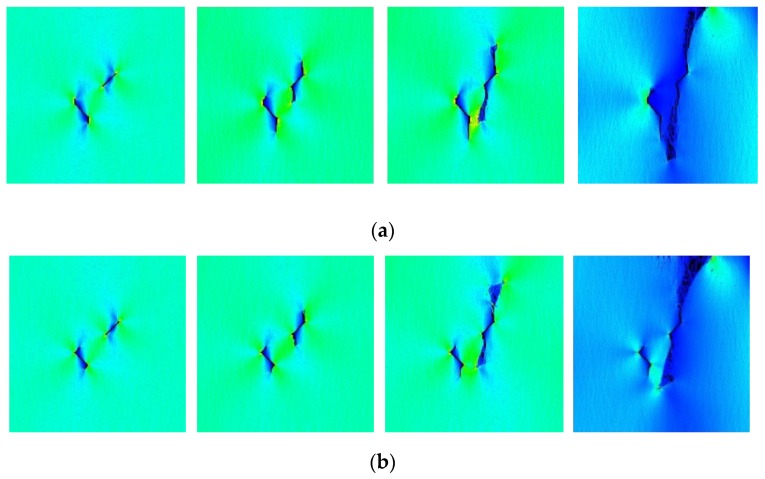
Sample failure mode II in which the rock bridge is not damaged. (**a**) Crack propagation process of sample KS - 45^#^; (**b**) Crack propagation process of sample KS - 90^#^.

However, the sample KS - 45^#^ only had tensile cracks that penetrated the entire sample, and the rock bridge was not damaged (Figure 14a). When the loading was continued for 20 steps, the crack tips of the two fissures initiated and formed tensile cracks. When the load continued to step 50, the tensile cracks extended along the axial direction. At step 60, the tensile cracks grow longer, and at step 80, tensile cracks penetrated through the whole sample while the rock bridge between two fissures was not damaged. The failure mode of sample KS - 90^#^ is similar to that of sample KS - 45^#^, as shown in Figure 14b. 

It can be seen from the simulation results that the results of the numerical simulations are closer to the actual experimental results, but they are not exactly the same. Their destruction paths are not the same, and they just have similar trends in destruction paths. Since the numerical simulation results cannot be completely consistent with the experiments, we only do a rough simulation of this. 

## 5. Conclusions

In this study, 3D printing technology was used to prepare the intermittent K–S fissure samples. Combined with the DIC experimental method, the strain field and failure modes around the K–S fissures under the uniaxial compression load were visually and quantitatively displayed. The main conclusions include the following:

- The kinked branches do not change the stiffness and strength of the samples too much. When the branch inclination angle of the kinked fissure rotates clockwise (β = +45°, +90°, or +135°), the peak strength increases. When the branch inclination angle of the kinked fissure rotates counterclockwise (β = −45°, −90°, or −135°), the peak strength does not change significantly, and the difference between the maximum and minimum strength does not exceed 10%.
- The primary crack may initiate from the inflection point or the end tip depending on the inclination angle β of the kink branch. When β = +45°, +90°, or +135°, the kinked fissures form crack from the inflection point. When β = −45°, −90°, or −135°), the kinked fissures incur crack from the end tip.
- The small kink branch may change the failure mode of the samples completely, and the inclination angle β of the kinked fissure has an important effect on the failure mode. When β = +45°, +90°, +135°, or –135°, a tension–shear composite failure mode (Mode I) occur in the rock bridge. When β = −45° and −90°, tensile failure is incurred throughout the whole samples, while the rock bridge does not damage.
- The numerical simulation failure modes of the models are well consistent with the cracking and failure modes of the physical experimental samples.

**Notions**:

I = Kinked fissure
II = Straight fissure
*θ*_1_*=* Angle between fissure I and the horizontal axis
*θ*_2_*=* Angle between fissure II and the horizontal axis
*β* = Angle between fissure II and the y-axis
2*a*_1_ *=* Main fracture length of fissure I
2*a*_2_ *=* Length of fissure II
2*c* = Distance between kinked fissure I and straight fissure II

## Figures and Tables

**Table 1:** Basic mechanical properties of 3D-printed samples.

Elastic Modulus (GPa)	Yield Strain (%)	Compressive Strength (Mpa)	Ultimate Strain (%)	Density (g/cm^3^)
2.7	3.3	60	11.0	1.12

**Table 2:** The basic mechanical properties of 3D-printed samples after freezing.

Poisson’s Ratio (*γ*)	Elastic Modulus (Gpa)	Tensile Strength (Mpa)	Compressive Strength (Mpa)
0.24	7.1	6.7	75

**Table 3:** 3D printed samples contain different fissures under uniaxial compression.

Number	Sample	*β*/°	Note
1	TS^#^		1. *θ*_1_ of all the samples is −45°; 2. *θ*_2_ of all the samples is +45°; 3. The length of *b* for the K–S fissures samples is 2 mm
2	KS + 45^#^	+45	
3	KS + 90^#^	+90	
4	KS + 135^#^	+135	
5	KS - 45^#^	−45	
6	KS - 90^#^	−90	
7	KS - 135^#^	−135	

Note: “TS” represent two straight fissures; “KS” represent a kinked fissure and a straight fissure; “+” represent rotating counterclockwise; “−” represent rotating clockwise.

**Table 4:** Summary of cracking behavior and coalescence patterns in the study.

Sample	Experimental Figures	Sketch of the Specimen	Cracking Pattern
TS^#^	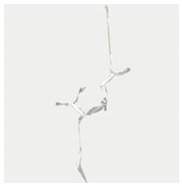	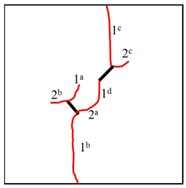	T: 1^a^, 1^b^, 1^c^, 1^d^; S: 2^a^; L: 2^b^,2^c^
KS + 45^#^	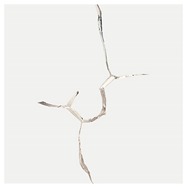	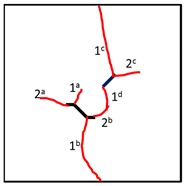	T: 1^a^, 1^b^, 1^c^, 1^d^; S: 2^b^; L: 2^a^,2^c^
KS + 90^#^	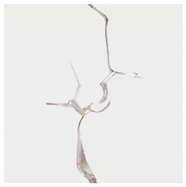	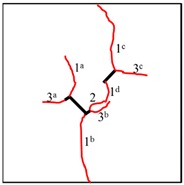	T: 1^a^, 1^b^, 1^c^, 1^d^; S: 2, 3^b^; L: 3^a^,3^c^
KS + 135^#^	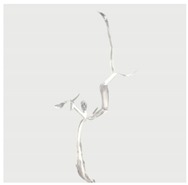	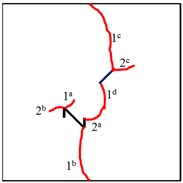	T: 1^a^, 1^b^, 1^c^, 1^d^; S: 2^a^; L: 2^b^, 2^c^
KS - 45^#^	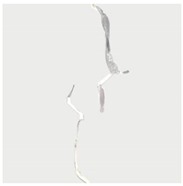	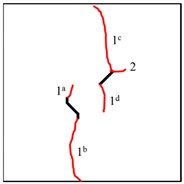	T: 1^a^, 1^b^, 1^c^, 1^d^; L: 2
KS - 90^#^	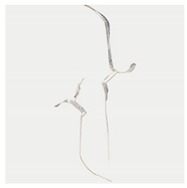	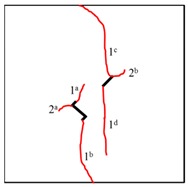	T: 1^a^, 1^b^, 1^c^, 1^d^; L: 2^a^, 2^b^
KS - 135^#^	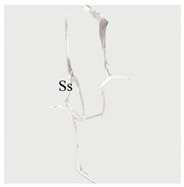	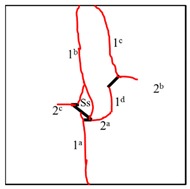	T: 1^a^, 1^b^, 1^c^, 1^d^; S: 2^a^; L: 2^b^, 2^c^

a, b, c, d represent one, two, three and four cracks in primary and secondary cracks each time.

**Table 5:** Mechanical parameters of the model element.

Internal Friction Angle (°)	Elastic Modulus (GPa)	Coefficient of Deformation	Press Pull Ratio	Poisson’s Ratio (*γ*)	Pull Coefficient	Density (g/cm^3^)
1.12	2.7	100	10	0.24	1.5	1.12
